# Communication networks beyond the capacity crunch

**DOI:** 10.1098/rsta.2015.0191

**Published:** 2016-03-06

**Authors:** A. D. Ellis, N. Mac Suibhne, D. Saad, D. N. Payne

**Affiliations:** 1 Aston University, Aston Triangle, Birmingham B4 7ET, UK; 2 University of Southampton, Highfield, Southampton SO17 1BJ, UK

**Keywords:** optical communications, capacity limits, mobile communications, energy

## Abstract

This issue of *Philosophical Transactions of the Royal Society, Part A* represents a summary of the recent discussion meeting ‘Communication networks beyond the capacity crunch’. The purpose of the meeting was to establish the nature of the capacity crunch, estimate the time scales associated with it and to begin to find solutions to enable continued growth in a post-crunch era. The meeting confirmed that, in addition to a capacity shortage within a single optical fibre, many other ‘crunches’ are foreseen in the field of communications, both societal and technical. Technical crunches identified included the nonlinear Shannon limit, wireless spectrum, distribution of 5G signals (front haul and back haul), while societal influences included net neutrality, creative content generation and distribution and latency, and finally energy and cost. The meeting concluded with the observation that these many crunches are genuine and may influence our future use of technology, but encouragingly noted that research and business practice are already moving to alleviate many of the negative consequences.

## 1. Introduction

The term ‘capacity crunch’ was first applied to communication networks at the start of the decade [1,2], and signalled the recognition that the transmission capacity of an optical fibre is not limitless. The relentless exponential growth in demand for data services, particularly video has, since 1975 [3], been largely fulfilled by using new technology to increase the capacity of a single optical fibre. This was viewed as a solid and predictable business opportunity by carriers, service providers and equipment manufacturers alike. However, once the maximum capacity of individual fibres is reached, coping with ever-increasing demand defaults to the age old ‘install more cables’ approach. This ‘post-crunch’ solution will have inevitable consequences for the business model of communication systems worldwide unless new approaches can be found to eke out the fibre capacity.

The terms ‘capacity crunch’ and ‘the nonlinear Shannon limit’ [4–6] (the physical limitation behind the capacity crunch itself) have been widely used in the scientific community as a rallying cry to develop innovative post ‘capacity crunch’ solutions. However, it should be noted that while in some fields such as oil exploration, the phrase capacity crunch has implied an impending exhaust of the available resources [7] (also known as ‘peak oil’), the more common use implies a finite and continuing, but restricted, ability to supply the resource [8,9]. It is in this latter context that ‘capacity crunch’ applies to optical communication networks, namely the continued existence of optical communication networks supporting much of modern society, economically, administratively and socially, but with limitations on availability.

The purpose of the Royal Society discussion meeting ‘Communication networks beyond the capacity crunch’, reported in this issue of *Philosophical Transactions A*, was to establish the time scales associated with the crunch, and to foresee how communication networks will evolve in the interim in order to meet the incessant demand for both wireless and fixed line. The meeting confirmed that, in addition to a capacity shortage within a single optical fibre [10–12], many other ‘crunches’ are foreseen, both societal and technical, including the following.

### (a) Technical limitations

— *Wireless spectrum*. While the vast majority of wireless connections are completed with optical fibre, the prevalence of wireless technology for end-user connection has resulted in severe congestion within the radiofrequency spectrum. This has led to calls to release spectrum from broadcast applications to support mobile services, despite the apparent spectral efficiency of a broadcast medium for simultaneous delivery of, for example, sporting events [13–15].
— *Common public radio interface (CPRI)*. Increasing the wireless carrier frequency to simultaneously increases individual user data access rates and to reduce cell sizes is placing increasing pressure on CPRIs. The use of directional and multiple-input–multiple-output radio systems, high-stability carrier frequency dissemination and high data rates is leading to individual interface rates which approach that of a single wavelength channel in the core of the optical network, significantly increasing costs [14].
— *Network management and switching*. The increasing diversity and intricacy of communication services results in a highly complex network that is difficult to reconfigure on short time scales in order to meet changing demand patterns. Current research is focusing on open source network management tools to facilitate greater flexibility in network resource sharing, and in radical new network designs combining previously separate functions [16–18].
— *Software*. Current network protocols were originally designed as an experimental testbed. It is remarkable that continued patches and updates have enabled these protocols, originally developed without the aid of contemporary software verification tools, to service worldwide communications demand. Given the widespread distribution of individual software elements, evolution towards a post-capacity crunch software platform seems more likely than a reboot to purpose-designed software [19].

### (b) Societal and economic influences

— *Network neutrality*. Part of the founding principle of that part of the communications infrastructure which we know as the Internet, net neutrality, was formalized in 2003 [20] and is currently subject to US legislative actions [21]. It relies on the principles of ‘dumb pipes’ which require over-provisioning of capacity. Its egalitarian aims assume that all content and services are of equal potential value and Internet operation on these principles has undoubtedly contributed to its success. However, the Internet is only one of several communication services carried by optical networks, and while the Internet is the dominant service, segregation (and in the context of net neutrality what some may call discrimination) of private line, voice and other data networks occurs. Furthermore, should the capacity crunch result in a decrease in the level of over-provisioning network suppliers are able (or willing) to offer, full network neutrality may become unsustainable.
— *Innovation within the creative industries*. Content delivery has rapidly developed from the early days of the Internet. Initially dominated by text-based traffic, then picture, today network video is being widely used by the creative arts and entertainment industry as a means of content delivery. Such content creators are constantly seeking new means of reaching the populous, such as virtual reality headsets that may further increase demand. Music collaborations in real time require ultra-low latency, and social media has evolved to the extent where content generation is distributed, requiring all users to have the bandwidth to upload high-definition video without delay. Future developments in the creative arts have the ability to create even further, as yet unforeseen, demand [22,23].
— *Latency*. As opposed to traditional consumption of services, the availability of sufficient connectivity to exchange audio and video files has led to a revolution in content generation, with vast quantities of data produced by individual users. Today, innovation within the creative industries aimed at collaborative endeavours is constrained by delays imposed by electronic switching and not by the volume of data. Coupled with the well-known latency demands of the gaming and financial industries, latency reduction is seen as a major benefit of any future post-capacity crunch network [24,25].
— *Energy*. As we approach the capacity crunch, the ability of the communication industry to reduce the energy used per bit of information with each successive product release is diminishing. Exponential demand growth will inevitably lead to rising energy consumption, with some of the costs borne by end users (who already provide power for the equipment on their premises). While decommissioning old, inefficient, equipment will partially mitigate this rise [26], the current level of around 1–2% of global energy consumption suggests that communications efficiency should form part of the decision-making processes for major endeavours [16].

Further details of many of these issues may be found in the subsequent articles. Here, we focus on three topics: the historical evolution of communication networks, illustrating a remarkable growth of nearly 15 orders of magnitude in capacity since the first use of fire beacons; the current optical fibre capacity crunch; and speculate on the possible future of communication networks where performance is constrained.

## 2. Capacity growth rates using electromagnetic fields

**Figure 1.**
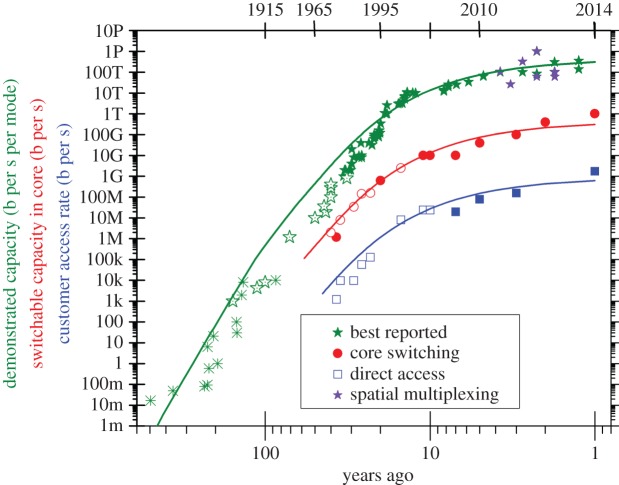
Evolution of communication capacity with time showing the best reported capacity including reports from scientific publications (green, purple), capacities switched within the core network from commercially available products (red) and data rates offered to residential customers in at least one country (blue). Symbols indicate use of free-space (crosses) or fibre (solid symbols) based optical systems or electronic systems (open symbols). Purple symbols represent spatial multiplexing; while curve fits assume that the compound annual growth rate is 5.75% times the global population in billions (figure 2) with the offset an arbitrary fitting parameter. Data taken from leading international conferences, manufacturers' data sheets and Internet resources.

Communication by light is well known, including direct human interaction and reading. We consider here the special case of communication beyond the unaided line of sight. Dating back thousands of years, early examples include communication beacons [27], where relay stations were used to transmit the message between consecutive stations. Relying on ignition or mechanical movements, message transmission was slow and messages transmitted were restricted to alarms. A considerable enhancement in performance was required before complex messages could be transmitted. This complexity may be added by increasing the rate at which signals are transmitted (for example smoke signals rather than simple ignition of a beacon), or by increasing the information content of each signal, enabling transmission of letters [28]. The overall system may be quantified by the product of the signal transmission rate (the symbol rate) and the complexity of each signal (measured in bits per symbol). Figure 1 illustrates (green points) the historical evolution of system capacity over the last 500 years. Crosses indicate free-space communication systems, including high transmission rates with the Murray shutter telegraph [29], and high symbol efficiency using flags [30], message duration and semaphore techniques. In this early period, exponential growth was observed (similar to Moore's law, which corresponds to a growth rate of 40% per annum), although the compound annual growth rates (CAGRs) were typically below 10%.

Free-space communication has many limitations that impact the maximum amount of information which may be transmitted, including environmental sensitivity (e.g. fog), the operating cost of manned relay stations and the susceptibility to crosstalk if multiple systems operate simultaneously. As communication demand began to exceed the capability of free-space optical communications, new systems based on transmission of signals over copper wires were introduced (open green stars in figure 1), commencing with the FiveWire Telegraph, which reflected the transmission of a letter for every symbol introduced with the Fryctoria system. This system was introduced in the UK in 1838 by Wheatstone and Cooke, but was eventually replaced with Morse's single wire system where code words based on binary symbols (e.g. Morse code) replaced the more efficient multi-bit symbols of the five wire system, but had the significant commercial advantage of requiring only a single wire for signal transmission. Growth rates increased to around 20% per annum, and the use of electronic signalling enabled significant cost reduction by the introduction of electronic amplifiers to replace human relay stations [31]. Within 50 years, transatlantic cables had been introduced, and soon covered the whole globe, reaching Adelaide, Australia in 1872.

Copper waveguides allowed capacities to grow for the next century, with the introduction of coaxial waveguides, frequency division multiplexing and multi-bit modulation such as quaternary phase shift-keying and orthogonal frequency division multiplexing. However, beyond systems capacities of about 1 Gbit s^−1^, the combination of propagation loss and bandwidth began to severely restrict deployment of higher-capacity systems, and once more a new transmission technology was required. Leading contenders were hollow metallic waveguides, operating with GHz carriers and the newly invented low loss fibre optic cables used to guide information encoded onto laser radiation.

Initial trials using multi-mode optical fibre [3,32] revealed competitive but not compelling bandwidths. Led by the British Post Office, the industry switched to single mode fibre (solid green stars in figure 1), offering the potential for substantially increased bandwidth, longer distances between electronic regenerators (replacing relay stations), and significantly lower size and weight. The case was compelling, and shortly after the first introduction of commercial fibre optic systems, transatlantic and transpacific systems were deployed. Following these initial installations, the immense capacity available predicated that development proceeded along economic grounds, initially using optoelectronic regenerators (relay stations) and binary modulation, and then, in turn, replacing relay stations with fixed capabilities with optical amplifiers, adding frequency (or wavelength) division multiplexing, introducing more complex coding to increase the information carried per symbol and allow for correction of errors, optimizing the detection technology and including quasi- or fully orthogonal frequency division multiplexing [33].

Today, physical limitations are once again becoming apparent, with systems designed according to fundamental limits introduced by noise (originating from quantum mechanical uncertainties in the number of photons added at each amplifier and ultimately limited by the Heisenberg uncertainty principle) and nonlinear distortions which are strongly dependent on the signal power [10–12,33]. Continuing demand for ever more and more communication bandwidth offers the prospect that demand may exceed the capacity limit imposed by these two features. This has been called the communications ‘capacity crunch’, and as we have seen, the approach of such incidents heralds not a collapse of communications infrastructure, but an opportunity for the introduction of revolutionary new technologies. Many new technologies may be considered, including signal processing [11] and new fibre optic waveguides [34], but the majority of industry insiders predict that it is likely that commercial pressures will dictate that the next dominant communication technology will be based on a re-introduction of space-division multiplexing [28], initially as parallel systems, but eventually, including direct spatial coding [35] and superchannels [36], and an optimization of the use of resources, including switching (or routing) of signals [17,18].

**Figure 2.**
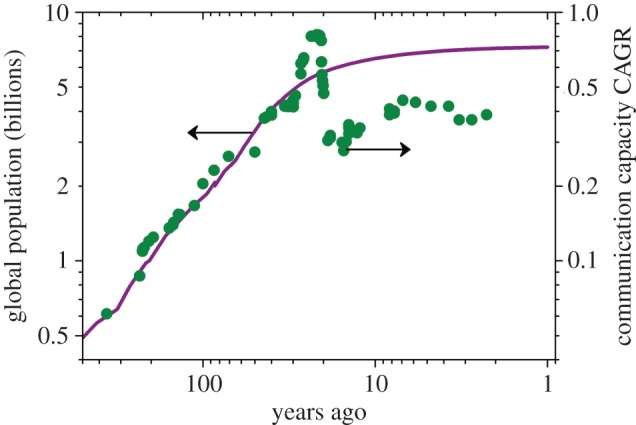
Evolution of global population (purple solid line), and compound annual growth rate (green filled circles, raw data smoothed over a minimum of 2 years) of communication capacity confirming correlation between the rate of increase of communication at a distance and population.

During the past 500 years, electromagnetic communications, in the guise of three revolutionary technologies (free-space optical, electronic and fibre-optic communications), have seen a remarkable and continuous growth, akin to Moore's law. Indeed, the CAGR over the past 20 years has been in the region of 40%, or doubling every 2 years, very close to the rate at which transistor densities are predicted to increase by Moore's law. It has enabled many significant enhancements to economic [37,38] and cultural growth [24,25], although occasionally fuelled (as many technologies have been) by military considerations. It is therefore perhaps interesting to examine correlations between society and communication capacity. One such comparison is shown in figure 2, where we plot the global population and the CAGR of communications capacity against time. Interestingly, the rapid expansion of population from 500 to 30 years ago correlates very strongly with the growth in communications capacity (the trend appears to continue further back in time to millennia, but verifiable data are sparse). Perhaps increasing population drives an increasing need to communicate; there are simply more people to talk to and more people to talk about. Equally, it may be due to an increasing ability to innovate as larger populations enable greater organization and the emergence of prodigious numbers of scientific specialists. Inspired by this correlation, the solid lines in figure 1 are calculated assuming a CAGR of 5.75% per billion of global population, showing excellent agreement.

Figures 1 and 2 also reveal a few additional features. Figure 2 shows a significant surge in available communication bandwidth growth rates between 1987 and 1995, corresponding to the period between the introduction of optical fibre to global submarine systems and the deployment of optical amplifiers [39]. Following this, there was a substantial correction and minimum growth rate around 2001. This oscillation, which is coincident with a point of inflection in population growth, appears to have foreshadowed the dot.com crash near the start of the millennium which induced major changes in the structure of the communications industry. Viewed over this 500 year time scale, both the communications capacity growth rate and the global population appear to have stabilized, with a CAGR for communications closer to 3% of global population corresponding to the widely reported 40% per annum [40]. Once again, causality is difficult to establish, but we note that the reduction in the proportionality constant between communications CAGR and global population follows the dot.com boom at the turn of the century. However, it should be noted that population and communications capacity both continue to be subject to exponential growth rates with correspondingly worrying implications for finite natural resources.

## 3. The optical fibre capacity crunch

We have seen above that our ability to communicate remotely has continuously improved over the last 500 years, and increases appear to correlate closely with global population. While possible, any significant reduction in the growth rate of communications traffic appears unlikely. However, as articles below will show, the maximum information flow through a fibre (or any communications channel) is finite. Greater information flow can be achieved by (i) increasing the channel spectral window, (ii) improving the spectral efficiency, or (iii) increasing the injected power to obtain greater signal-to-noise ratio at the receiver, all of which have limits.

The latter at first sight might appear the most attractive, but apart from the obvious limit when a typical telecom fibre self-destructs at a few tens of watts owing to dielectric breakdown, there are subtle implications of the nonlinear Kerr effect that introduce crosstalk between communication channels and appear well before this. The power dependence of the Kerr effect results in an optimum (much lower) signal power and a predicted maximum information spectral density [5,10–12,33]. There has been considerable research into the effect based on approximating or integrating the nonlinear Schrödinger equation under a variety of different approximations. These include (i) exact calculation of the interaction efficiency as a function of transmission distance and frequency for a periodically amplified system and integrating the noise induced by this interaction, (ii) Gaussian noise approximations, and (iii) perturbation analysis. The evolution of this field may be readily traced [33], and a consensus has emerged.

While representing only a lower bound on the fibre capacity [10], considering a nonlinear signal-to-noise ratio has proven fruitful for predicting overall system performance. At low launch powers, the signal-to-noise ratio is dominated by the amplified spontaneous emission which may be readily calculated from amplifier input powers, gains and noise figures. For higher launch powers, nonlinear noise is introduced via the Kerr nonlinearity. The nonlinear interaction in a centrosymmetric medium is third-order, and many presentations of the nonlinear signal-to-noise ratio are presented as cubic in power. They scale as the nonlinear coefficient squared, and inversely with loss (effective length) and dispersion (which weakens phase matching). While slightly more exact functions are available, the scaling parameter used to determine the nonlinear signal-to-noise ratio for a wavelength division multiplexed system with a uniform signal power distribution across a bandwidth *B* for a channel detuned in frequency from the centre of the band (given by *ω*_0_/2*π*) by *Δf* is given by
3.1
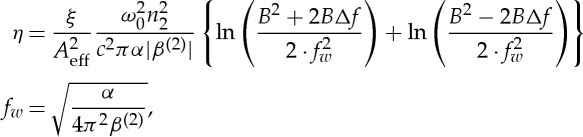
where *c* is the speed of light and *n*_2_, *A*_eff_, *α*, *β*^(2)^ and *ξ* are the fibre nonlinear refractive index, effective area, loss coefficient, group velocity dispersion and a parameter representing the impact of mode averaging in birefringent fibres, respectively [41,42]. It may then be shown that the nonlinear signal-to-noise ratio may be approximated to
3.2
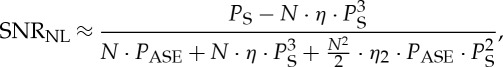
where *P*_S_ and *P*_ASE_ are the power spectral densities per polarization of the signal launched into each span and the amplified spontaneous emission added by each amplifier, respectively. The strength of the parametric noise amplification *η*_2_ is identical to *η* in the absence of nonlinearity compensation [43]. The first terms in the numerator and denominator represent the linear signal-to-noise ratio, and with *η*=*η*_2_=0, equation (3.2) gives the conventional Shannon limit. The second and third terms in the denominator represent interchannel nonlinearities [44] and parametric noise amplification [43], respectively, whereas the second term in the numerator represents the transfer of energy from the signal to the other channels [44,45]. The final terms in both numerator and denominator are generally neglected, unless efficient nonlinear compensation is employed [11].

**Figure 3.**
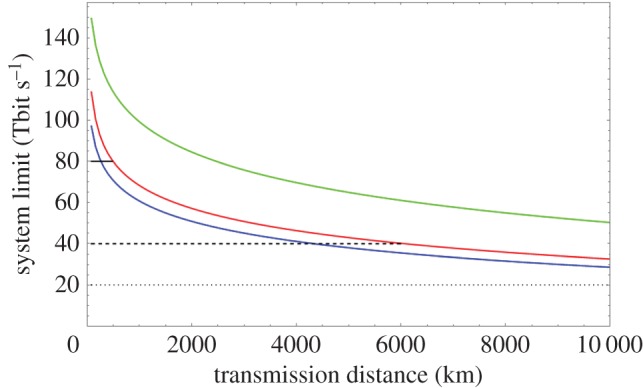
Maximum system throughput as a function of transmission distance assuming a 5 THz total bandwidth without compensation of nonlinearity (blue), with ideal compensation of nonlinearity based on digital signal processing (green) and with compensation of nonlinearity constrained by polarization mode dispersion (red). Horizontal lines show reach limits for polarization multiplexed QPSK (dot), 16QAM (dashed) and 256QAM (solid).

Figure 3 shows the maximum potential capacity using conventional erbium-doped fibre amplifiers (5 THz bandwidth) spaced every 80 km. It shows the relative performance of systems without any compensation of the nonlinearity (blue), with ideal compensation (*η*=0, green) and assuming that the nonlinear compensation is degraded by polarization mode dispersion (PMD) [46]. For the impact of PMD, we assume a coefficient of 0.02 ps km^−1/2^ corresponding to the minimum values observed for fibres spun during fabrication [47]. Optical superchannels are typically used to maximize the system capacity by maximizing the utilization of the available spectrum, and may readily be based on symbol rates in the range of 25–33 Gbaud. In this context, while the reach of today's 100 Gbit s^−1^ channels (polarization multiplexed quadrature phase-shift keying (QPSK)) is sufficient for the majority of applications and 200 Gbit s^−1^ channels (polarization multiplexed 16 state quadrature amplitude modulated (PM 16 QAM)) the majority of terrestrial applications and submarine applications with feasible compensation of nonlinearity, operation of 400 Gbit s^−1^ channels (PM 256QAM) appears confined to niche applications, unless the deleterious effects of PMD on the efficiency of nonlinearity compensation may be addressed.

Commercial systems are now available with 200 Gbit s^−1^ based superchannels, and while systems are not currently deployed fully populated there appears to be little remaining scope to increase the capacity per channel by any significant factor without major innovations in nonlinear optical transmission techniques that also compensate for the stochastic signal variations caused by PMD and noise.

## 4. Beyond the capacity crunch

Human nature and historical growth rates that appear to be saturating at a CAGR of 40% per annum suggest that demand for greater communication capacity will continue for the foreseeable future. On the other hand, nonlinear effects and/or power handling do impose a fundamental limit on the capacity of both optical fibre and wireless communication channels. Recent work has shown that the nonlinearity imposed by the Kerr effect may be compensated [11,12,33], but only up to the limit imposed by the nonlinear interaction between signal and noise. Theoretical work has shown that even this may be partially compensated by distributing optical phase conjugation devices [43] or optical regenerators [47] along the transmission link. However, this does not improve the simple power limit imposed by fibre damage and eventually new solutions will be required. For both the mobile and fibre sections of the network, space division multiplexing (SDM) appears to be the only remaining solution. Here, one may observe parallels with the field of computing, where the tailing increase in clock speed and device densities require new solutions for increasing computing power in the form of multicore processors, computer clusters and new architectures. Potentially, at the network level, a more holistic approach that better exploits the time-dependent communication patterns and demand could be employed to optimize the use of resource, with possible deviation from the current policy of network neutrality.

For optical fibre networks, an SDM solution may be as simple as installing many parallel cables/fibres, which sounds perfectly reasonable. However, while the communications industry has historically always reduced the energy consumption per bit with almost every new technology deployed [48] to meet growing consumer capacity demands, it is difficult to see that this trend can be maintained if additional copies of the existing technologies are deployed. Recent industrial- and government-funded (such as MODE-GAP and EXAT) projects have studied a wide range of alternative fibre types offering the prospect of performance above and beyond what is achievable from using an equivalent number of standard single mode fibres [2,34]. These fibres have included few-mode and multi-mode fibres, multicore fibres, hollow core fibres and various combinations of these. While no general consensus has yet been reached, it is apparent that the power consumption from optical amplification and digital signal processing for few-mode and multi-mode fibres will not be substantially lower than parallel single mode fibres. Such fibres may alleviate the capacity crunch, especially in applications where space for cabling is severely constrained, but will do little to reduce the burgeoning energy consumption of communications. Consequently, in the immediate future, novel fibres are most likely to find applications in niche markets where different constraints apply, such as collaborative content generation [25] and finance, where low latency is critical (hollow core fibres) and data centres where cable space is a premium (multicore or few-mode fibres). However, this could change if very-low nonlinearity fibres (e.g. hollow core) could also have a loss similar to or lower than existing telecom fibres, thus overcoming the nonlinear Kerr limit and simultaneously requiring lower injected power.

It is our view that it is within the core network where any capacity crunch would first be felt, spatial multiplexing solutions are just as likely to employ parallel strands of standard single mode fibre as novel fibre types. However, rather than lighting up many parallel systems, it is clear that demands to achieve significant per bit cost and energy consumption reductions will require future SDM systems to have certain key characteristics, including:

— *Photonic integration of transmitters and receivers.* These photonic-integrated circuits, fabricated monolithically or using hybrid technologies in traditional optoelectronic material systems or using a form of silicon photonics designed to exploit otherwise obsolete CMOS fabrication plants, will reduce footprint and packaging costs. This simplifies requirements for thermal stabilization, while allowing cooperative modulation, digital signal processing and coding over multiple spatial or frequency paths. Multiple client side interfaces will allow network side traffic to be routed over one of more diverse spatial paths.
— *Spatially multiplexed (multimode/multicore/ribbon) fibres* will provide parallel links with known and stable differences in propagation characteristics that will interface readily with existing non-spatially multiplexed regions of the network and will provide orders of magnitude increases in capacity over the nonlinear Shannon limit.
— *Spatially multiplexed optical amplifiers* will allow the overhead associated with installation, control, management and pump redundancy to be shared over multiple spatial paths, reducing the overhead from a minimum 1:1 provisioning to as little as 1:*n* provisioning, depending on application reliability specifications. They will be usable with both conventional networks and with at least one type of spatially multiplexed fibre type.
— *Network nodes* will be implemented supporting a significantly higher number of spatial and spectral paths, providing cost reduction through reduced component count, joint switching of spatial–spectral superchannels and the ability to groom traffic within superchannels optically.

In isolation, each of the SDM-motivated innovations would offer specific advantages to network operators and would enable some aspect of costs to be managed. However, when used fully in combination with each other, significant network-level benefits may also be anticipated. These advantages include (i) reduced blocking owing to the availability of alternative spatial paths for a given wavelength and consequently simplified resource allocation and network management, (ii) increased flexibility, (iii) increased transparent (regenerator-free) reach, and (iv) traffic grooming in the optical layer allowing the number of core router ports to be reduced significantly. Taking into account that the likely customer bandwidth demands in a post-capacity crunch network (extrapolate figure 1 at 40% per annum) will match or exceed that of a single wavelength channel, it is even conceivable that routers in the core network could be bypassed completely.

It is of course conceivable that post-capacity crunch, the difficulties associated with exponentially increasing traffic demands will lead to changes in the market dynamics of our communication networks, with service providers or consumers paying network operators directly or indirectly for actual bandwidth use. It is equally conceivable that, after many centuries of building higher capacity networks and then discovering new applications, our ability to communicate remotely has finally matched our ultimate desires, and that CAGRs above 10% were a feature of the twentieth century. However, while there are some signs of necessary and overdue movement in the economic financing of communication networks, the authors see little evidence that demand is beginning to saturate. We continue to be astounded by the innovative applications that appear when end users and early adopters are provided with an oversupply of capacity for current applications. Others are of the opinion that capacity should only be delivered on a needs–must basis, that is, once demand for an application is certain. This debate has bubbled away for many decades. Historically, ‘build it and they will come’ has proven to be a highly successful strategy, but as many investors state, past performance is no guide to the future. The debate will therefore inevitably continue until the day that the cautious view prevails, irrespective of any amount of historical precedent.

## 5. Conclusion

Communication networks now form an integral part of our daily lives, our national economy and social interaction. Connections to the network are increasingly through wireless devices, but nearly all signals are transmitted over optical fibres at some point along their route. As may be seen throughout this discussion meeting issue, the existence of a capacity crunch is certain, and impacts many aspects of our communication networks simultaneously. While the precise timing of each individual crunch is a matter for speculation, well-known techniques of scarce resource management are increasingly likely to be deployed in communication networks. To ease the transition from the resource-rich environment of the last 20 years, many innovative solutions are currently being investigated, and a selection may be found in the following pages. Consequently, while some modest changes may be expected, communication networks built on optical fibres are set to remain a central pillar of modern society for the foreseeable future.

## References

[1] Chraplyvy A 2009 The coming capacity crunch. In *Proc. 35th European Conf. on Optical Communications,**Vienna, Austria, 20–24 September 2009*. See http://ieeexplore.ieee.org/stamp/stamp.jsp?tp=&arnumber=5287305&isnumber=5286960.

[2] Richardson DJ 2010 Filling the light pipe. *Science* 330, 327–328. (10.1126/science.1191708) 20947751

[3] The Editor. 1975 First non-military fibre-optic link. *Electron. Power* 22, 285 (10.1049/ep.1976.0127)

[4] Antona J-C 2009 Key technologies for present and future optical networks. In *Proc. TWEPP-09, Topical Workshop on Electronics for Particle Physics*, *Paris, France, 21–25 September 2009*. See https://indico.cern.ch/event/49682/contribution/154.

[5] Ellis AD 2010 Approaching the non-linear Shannon limit. *J. Lightwave Technol.* 28, 423–433. (10.1109/JLT.2009.2030693)

[6] Tkach RW 2010 Network traffic and system capacity: scaling for the future. In *36th European Conf. on Optical Communication,**Turin, Italy, 19–23 September 2010*. Paper number We.7.D.1 . (10.1109/ECOC.2010.5621214)

[7] Stephens P 2009 *The coming oil supply capacity crunch*. A Chatham House Report. London, UK: Chatham House.

[8] Butler M 2005 Animal cell cultures: recent achievements and perspectives in the production of biopharmaceuticals. *Appl. Microbiol. Biotechnol.* 68, 283–291. (10.1007/s00253-005-1980-8) 15834715

[9] Conti JP 2015 Airport of the future. *Eng. Technol.* 10, 66–68. (10.1049/et.2015.0132)

[10] Agrell E, Alvarado A, Kschischang FR 2016 Implications of information theory in optical fibre communications. *Phil. Trans. R. Soc. A* 374, 20140438 (10.1098/rsta.2014.0438) 26809578

[11] Bayvel P Submitted Maximising the optical network capacity. 10.1098/rsta.2014.0440 PMC4733919 26809572

[12] Essiambre R, Kramer G, Winzer PJ, Foschini GJ, Goebel B. 2010 Capacity limits of optical fiber networks. *J. Lightwave Technol* **28**, 662–701. (10.1109/JLT.2009.2039464)

[13] Poularakis K, Tassiulas L. 2016 Cooperation and information replication in wireless networks. *Phil. Trans. R. Soc. A* 374, 20150123 (10.1098/rsta.2015.0123) 26809574

[14] Chih-Lin I, Han S, Xu Z, Sun Q, Pan Z 2016 5G: rethink mobile communications for 2020+. *Phil. Trans. R. Soc. A* 374, 20140432 (10.1098/rsta.2014.0432) 26809577

[15] Chang G-K, Cheng L 2016 The benefits of convergence. *Phil. Trans. R. Soc. A* 374, 20140442 (10.1098/rsta.2014.0442) 26809570

[16] Kilper DC, Rastegarfar H 2016 Energy challenges in optical access and aggregation networks. *Phil. Trans. R. Soc. A* 374, 20140435 (10.1098/rsta.2014.0435) 26809581

[17] Nejabati R, Peng S, Simeonidou D 2016 Optical network democratization. *Phil. Trans. R. Soc. A* 374, 20140443 (10.1098/rsta.2014.0443) 26809571

[18] Rofoee BR *et al* 2013 Demonstration of low latency intra/inter data-centre heterogeneous optical sub-wavelength network using extended GMPLS-PCE control-plane. *Opt. Express* **21**, 5463–5474. (10.1364/OE.21.005463) 23482117

[19] Zilberman N, Moore AW, Crowcroft JA. 2016 From photons to big-data applications: terminating terabits. *Phil. Trans. R. Soc. A* **374**, 20140445. (10.1098/rsta.2014.0445) PMC4733921 26809573

[20] Wu T 2003 Network neutrality, broadband discrimination. *J. Telecommun. High Technol. Law* 2, 141.

[21] Weinberg M 2014 Landmark day for net neutrality. *Public Knowledge*, 15 September 2014.

[22] Ha K, Pillai P, Lewis G, Simanta S, Clinch S, Davies N, Satyanarayanan M 2013 The impact of mobile multimedia applications on data center consolidation. In *IEEE Int. Conf. on Cloud Engineering (IC2E),**Redwood City, CA, 25–27 March 2013*, pp. 166–176 (10.1109/IC2E.2013.17)

[23] Barker SK, Shenoy P 2010 Empirical evaluation of latency sensitive application performance in the cloud. In *Proc. 1st Annual ACM SIGMM Conf. on Multimedia Systems*, pp. 35–46. New York, NY: ACM (10.1145/1730836.1730842)

[24] Chambers CJ 2016 Future traffic demands and characteristics from a media perspective. *Phil. Trans. R. Soc. A* 374, 20140433 (10.1098/rsta.2014.0433) 26809579

[25] Mansell R, Foresta D 2016 Social value of high bandwidth networks: creative performance and education. *Phil. Trans. R. Soc. A* 374, 20150124 (10.1098/rsta.2015.0124) 26809576

[26] Lord A, Soppera A, Jacquet A 2016 The impact of capacity growth in national telecommunications networks. *Phil. Trans. R. Soc. A* 374, 20140431 (10.1098/rsta.2014.0431) 26809568

[27] Homer, *The Iliad*. Wordsworth Editions Limited, 2003.

[28] Universal Postal Union. www.upu.int See http://www.upu.int/en/the-upu/history/about-history.htm (accessed 23 June 2015).

[29] MacKechnie Jarvis C 1956 The history of electrical engineering. Part 5: The origin and development of the electric telegraph. *J. Inst. Electr. Eng*. 2, 130–137. (10.1049/jiee-3.1956.0054)

[30] Popham HR 1801 Telegraphic signals or marine vocabulary 1801. (Transcribed by P. Ball). London, UK: C. Roworth.

[31] Loring AE 1878 A hand-book of the electro-magnetic telegraph, pp. 53–54. New York, NY: D. Van Nostrand.

[32] Li T 1978 Optical fiber communication—the state of the art. *IEEE Trans. Commun*. 26, 946–955. (10.1109/TCOM.1978.1094175)

[33] Kumar S (ed.) 2011 Impact of nonlinearities on fiber optic communications. New York, NY: Springer.

[34] Richardson DJ 2016 New optical fibres for high-capacity optical communications. *Phil. Trans. R. Soc. A* 374, 20140441 (10.1098/rsta.2014.0441) 26809569 PMC4733920

[35] Eriksson TA, Luís RS, Puttnam BJ, Mendinueta JMD, Andrekson PA, Karlsson M, Awaji Y, Wada N, Agrell E 2015 Single parity check-coded 16QAM over spatial superchannels in multicore fiber transmission. *Opt. Express* 23, 14 569–14 582. (10.1364/OE.23.014569) 26072817

[36] Klonidis D *et al* 2015 Spectrally and spatially flexible optical network planning and operations. *IEEE Commun. Mag*. 53, 69–78. (10.1109/MCOM.2015.7045393)

[37] Jorgenson DW, Ho MS, Samuels JD, Stiroh KJ 2007 Industry origins of the American productivity resurgence. *Econ. Syst. Res*. 19, 229–252. (10.1080/09535310701571885)

[38] Kavesh RA, Garbade KD, Silber WL 1978 Technology, communication and the performance of financial markets: 1840–1975. *J. Fin*. 33, 819–832. (10.1111/j.1540-6261.1978.tb02023.x)

[39] Abbott S 2008 Review of 20 years of undersea optical fiber transmission system development and deployment since TAT-8. In *34th European Conf. on Optical Communication, Brussels, Belgium, 21–25 September 2008* (10.1109/ECOC.2008.4729092)

[40] Cisco Visual Networking Index. See http://www.cisco.com/c/en/us/solutions/service-provider/visual-networking-index-vni/index.html.

[41] Chen X, Shieh W 2010 Closed-form expressions for nonlinear transmission performance of densely spaced coherent optical OFDM systems. *Opt. Express* 18, 19 039–19 054. (10.1364/OE.18.019039) 20940798

[42] Ellis AD, Mac Suibhne N, Sygletos S, Garcia Gunning FC 2013 Expressions for the nonlinear transmission performance of multi-mode optical fiber. *Opt. Express* 21, 22 834–22 846. (10.1364/OE.21.022834) 24104170

[43] Ellis AD, McCarthy ME, Al-Khateeb MAZ, Sygletos S 2015 Capacity limits of systems employing multiple optical phase conjugators. *Opt. Express* **23**, 20 381–20 393. (10.1364/OE.23.020381)

[44] Mitra PP, Stark JB 2001 Nonlinear limits to the information capacity of optical fibre communications. *Nature* 411, 1027–1030. (10.1038/35082518) 11429598

[45] Poggiolini P, Carena A, Jiang Y, Bosco G, Curri V, Forghieri F 2014 Impact of low-OSNR operation on the performance of advanced coherent optical transmission systems. In *European Conf. on Optical Communications, Cannes, France, 21–25 September 2014* (10.1109/ECOC.2014.6964096)

[46] IEC 60794-3, September 2001 (N=20, Q=0.01%). Details are described in IEC 61282-3 TR Ed 2, October 2006.

[47] Wabnitz S, Eggleton B (eds). 2015 *All optical signal processing*. Berlin, Germany: Springer.

[48] Tucker RS 2011 Green optical communications. I. Energy limitations in transport. *J. Select. Top. Quantum Electron*. 17, 245–260. (10.1109/JSTQE.2010.2051216)

